# Applications of Chitosan in Surgical and Post-Surgical Materials

**DOI:** 10.3390/md20060396

**Published:** 2022-06-15

**Authors:** Fernando Notario-Pérez, Araceli Martín-Illana, Raúl Cazorla-Luna, Roberto Ruiz-Caro, María Dolores Veiga

**Affiliations:** Department of Pharmaceutics and Food Technology, Faculty of Pharmacy, Universidad Complutense de Madrid, Plaza Ramón y Cajal s.n, 28040 Madrid, Spain; fnotar01@ucm.es (F.N.-P.); aracelimartin@ucm.es (A.M.-I.); racazorl@ucm.es (R.C.-L.); rruizcar@ucm.es (R.R.-C.)

**Keywords:** bleeding control, bone regeneration, hemostasis, hydrogels, scaffolds, soft tissue, sponges, sutures, tissue regeneration, viscosupplementation

## Abstract

The continuous advances in surgical procedures require continuous research regarding materials with surgical applications. Biopolymers are widely studied since they usually provide a biocompatible, biodegradable, and non-toxic material. Among them, chitosan is a promising material for the development of formulations and devices with surgical applications due to its intrinsic bacteriostatic, fungistatic, hemostatic, and analgesic properties. A wide range of products has been manufactured with this polymer, including scaffolds, sponges, hydrogels, meshes, membranes, sutures, fibers, and nanoparticles. The growing interest of researchers in the use of chitosan-based materials for tissue regeneration is obvious due to extensive research in the application of chitosan for the regeneration of bone, nervous tissue, cartilage, and soft tissues. Chitosan can serve as a substance for the administration of cell-growth promoters, as well as a support for cellular growth. Another interesting application of chitosan is hemostasis control, with remarkable results in studies comparing the use of chitosan-based dressings with traditional cotton gauzes. In addition, chitosan-based or chitosan-coated surgical materials provide the formulation with antimicrobial activity that has been highly appreciated not only in dressings but also for surgical sutures or meshes.

## 1. Introduction

Improvement in health care systems has led to a notable increase in expected lifetimes and standard of living [1]. It is a truism that surgery is nowadays an important factor in improving people’s health. Differences in the state of surgical care across countries and among people from various socioeconomic standings have been clearly related to inequalities in levels of death and disability. Even easily treatable problems become serious illnesses when safe and accessible surgical care is not available. Thus, it is estimated that in 2010, around 16.9 million people died due to a lack of access to surgical care [2]. Therefore, surgical care must be understood to be a central part of health systems and strategies to improve the standard of living.

Access to surgical care, as well as the quality of surgical conditions and post-surgical follow-up, is important since complications from surgical procedures can also lead to serious disabilities. Post-operative wound complications, such as wound bleeding, serum fluid accumulation, or wound dehiscence, are quite common and may derive from surgery- or patient-related causes [3]. Another frequent and serious complication is surgical site infections—associated with 2–11% of surgical interventions—which cause an increase in treatment costs and mortality [4]. Proper post-operative antibiotic prophylaxis can significantly reduce the rate of infection in both clean and contaminated wounds. Hemorrhagic and thrombotic complications can also occur during or after a surgical procedure. They can be caused by patient-related hemostatic functional problems or by improper closure of blood vessels during surgery. Early detection and proper treatment of these complications are crucial to avoid a drastic outcome [5].

In recent decades, there has been an increase in the quality of surgical care, mainly in terms of the techniques and materials used in surgical procedures. In this sense, biopolymers have emerged as a valuable tool for surgical materials and the management of post-surgical complications. These biopolymers are natural macromolecules that can be obtained from plant, animal, or microbial sources and, generally, stand out for their biocompatibility [6]. Collagen, hyaluronic acid, cellulose, and alginate are examples of biopolymers widely used in materials for surgical procedures. In addition to their biocompatibility, the so-called functional polymers can also provide several advantages to the developed systems, such as support for cell growth or antimicrobial, anti-inflammatory, and adhesive properties.

Chitosan is one of those biopolymers that has gained importance as a material used in surgical procedures. It is a non-toxic polysaccharide that is a valuable option for fabricating devices for surgical applications due to its biocompatibility and biodegradability [7]. Various chitosan-based products, such as scaffolds [8], hydrogels [9], surgical glues [10], or implants [11], have been developed for surgical applications. Moreover, its great versatility and the functional properties it provides make this biopolymer useful for a wide variety of applications. Thus, the inherent microbicidal and adhesive properties of chitosan have been exploited in several ways. Some examples include the use of chitosan as an adhesive and antimicrobial material [12], as a hemostatic agent [13], for tissue repair [11], and for wound healing and tissue engineering [6]. Finally, chitosan-based modified polymers have also been developed to provide additional benefits to these materials. The limited solubility of the polymer in neutral and alkaline pH is a limitation that can be overcome by chemical modifications, such as the thiolation or acylation of chitosan [14]. This review offers a critical view of the latest surgical advances in chitosan-based biomaterials and their applications to surgical care. Chitosan-based therapies intended for post-surgical prophylactic or palliative treatment have also been considered.

## 2. Properties of Chitosan

Chitosan is one of the most studied polysaccharides in recent decades for its biomedical application. This polymer is derived from chitin, the main component in the exoskeleton of insects and crustaceans, a homopolymer consisting of β-(1→4)-N-acetyl-D-glucosamine [15,16]. The partial deacetylation of chitin in an alkaline medium allows us to obtain chitosan, a heteropolymer composed of N-acetyl-D-glucosamine-β-(1→4)-D-glucosamine [17,18], with an average molecular weight of 100–500 kDa. The degree of deacetylation of chitosan depends on the conditions applied during the deacetylation process—such as temperature or sodium hydroxide concentration—and determines various properties of the polymer, such as pKa, solubility, and viscosity [18].

**Figure 1:**
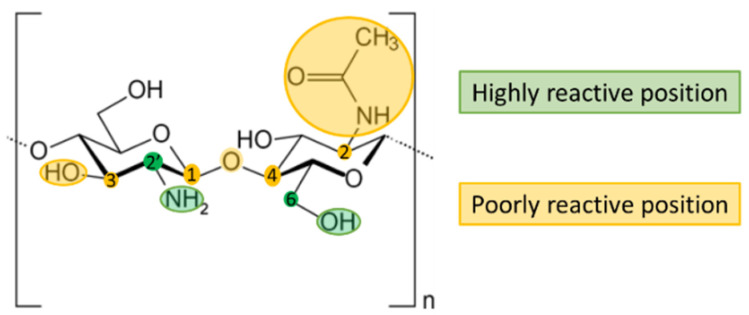
Chemical structure of chitosan. Highly reactive positions are highlighted in green; poorly reactive position are highlighted in yellow. Reprinted from [21] under the terms of the Creative Commons Attribution License 4.0 (copyright 2021 Cazorla-Luna et al.; doi:10.3390/polym13142241).

The chitosan molecule includes hydroxyl groups (C3 and C6), amine groups (C2′), acetamide groups (C2), and O-glycosidic bonds (C1-C4) (Figure 1). The acetamide and O-glycosidic bonds are highly stable and, therefore, poorly reactive, as is the hydroxyl group at C3 since it is a secondary hydroxyl with a high steric hindrance. On the contrary, either the hydroxyl group at C6 or the primary amine at C2′ can be exploited to include different functional groups, thus modifying the physicochemical properties of the molecule [19]. Taking advantage of this characteristic, different modifications of chitosan have been proposed to date, including N-alkylation or O-alkylation to obtain carboxymethyl chitosan and N-acylation with glutamic acid, phosphorylation, or sulfonation. Consequently, the different derivatives also exhibit different properties: for instance, carboxymethyl chitosan shows enhanced film-forming and bacteriostatic properties, phosphorylated chitosan promotes bone regeneration and calcium absorption, and sulfonated chitosan has anticoagulant effects [20].

Chitosan is insoluble in neutral or alkaline media but soluble in acid media due to the protonation of its primary amine. This makes chitosan a unique biopolymer as it is the only high-weight cationic biopolymer known to date. Among the most frequent diluted acids used to dissolve chitosan, acetic acid, formic acid, hydrochloric acid, and lactic acid can be highlighted, while sulfuric acid and phosphoric acid do not dissolve chitosan [22,23]. Its positive charge in acid media allows chitosan to interact with polyanions through electrostatic interactions [15,21]. In addition, protonated amino groups can interact with the sialic groups of mucin, conferring this polymer the ability to bind mucosal surfaces, directly depending on the degree of deacetylation [16,24,25].

Chitosan is biocompatible, biodegradable, non-toxic, and non-immunogenic. It has several properties, such as being bacteriostatic, fungistatic, hemostatic, analgesic, and antiulcerous [16,22]. Due to these properties, chitosan and its derivatives have been widely explored as an excipient in different healthcare materials. To begin with, chitosan has been used for the development of different drug delivery systems, including mucoadhesive, multiparticulate parenteral, and floating oral drug delivery systems [26,27,28,29,30]. Moreover, chitosan has been explored for tissue engineering, sutures, and wound dressings, taking advantage of its bacteriostatic and healing properties [31,32,33,34]. Other applications include the development of biosensors [35].

## 3. Chitosan-Based Materials and Devices

**Figure 2:**
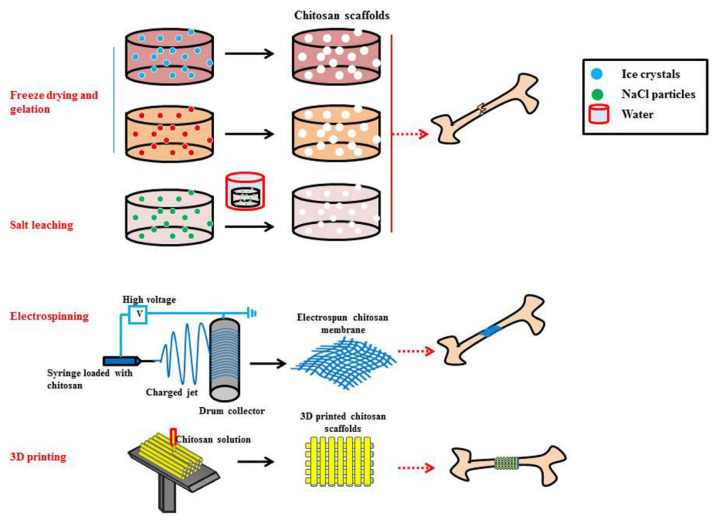
Schematic representation of the commonly used fabrication methods for producing chitosan-based scaffolds—freeze-drying, freeze gelation, salt leaching, electrospinning, and 3D printing. Reprinted with permission from Saravanan et al. [36]. Copyright 2016, Elsevier.

Chitosan has been used in a broad assortment of medical materials and devices (Figure 2). The most common are described below. Each system benefits from the properties (described above) that chitosan can provide for surgical applications. The shape, porosity, consistency, and size of the fabricated systems can be precisely tuned for the intended application.

### 3.1. Scaffolds

**Figure 3:**
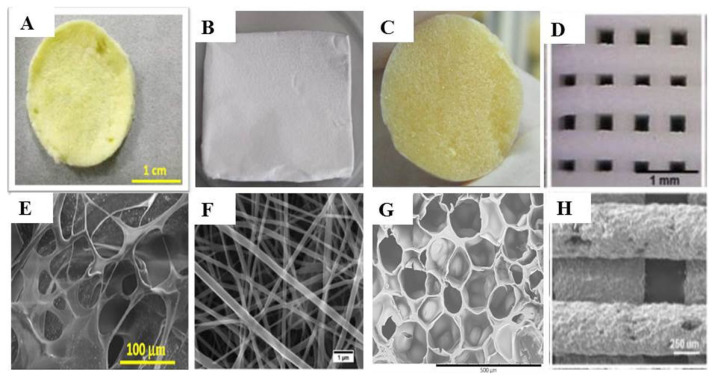
Macroscopic and microscopic images of scaffolds fabricated by different methods in bone tissue engineering. (**A**–**D**) show the macroscopic images of scaffolds prepared by freeze-drying, electrospinning, the sol–gel method, and 3D-bioprinting, respectively, and (**E**–**H**) show the corresponding SEM images of the scaffolds. Reprinted with permission from Soundarya et al. [42]. Copyright 2018, Elsevier.

Scaffolds are porous solids with controlled geometry and microstructures (Figure 3). They provide extracellular support for cell proliferation and can also serve as a template, for example, to guide tissue regeneration [37]. Biocompatible polymers such as chitosan are considered for the manufacture of implantable scaffolds since they generally ensure absorption/degradability capacity and the absence of toxicity [38]. A described method for the fabrication of chitosan-based scaffolds is the lyophilization of chitosan gels and solutions [39]. This technique makes it possible to control the average pore diameter—which varies from 1 to 250 μm—by means of freezing conditions. An alternative is the manufacturing of chitosan scaffolds by 3D printing, a technique that allows us to obtain systems with a tightly controlled shape and structure [40]. Finally, it is also possible to obtain self-assembled scaffolds. For this, it is necessary to use a second raw material that, when combined with chitosan, spontaneously forms a scaffold structure. An example is the manufacture of hybrid scaffolds of chitosan and sericin; the positive charges present in the chitosan structure react with the negative charges of aspartic and glutamic acids that are present in the serine structure [41]. This combination also improves cell adhesion and porosity, maintaining the biocompatibility of the system.

### 3.2. Sponges

**Figure 4:**
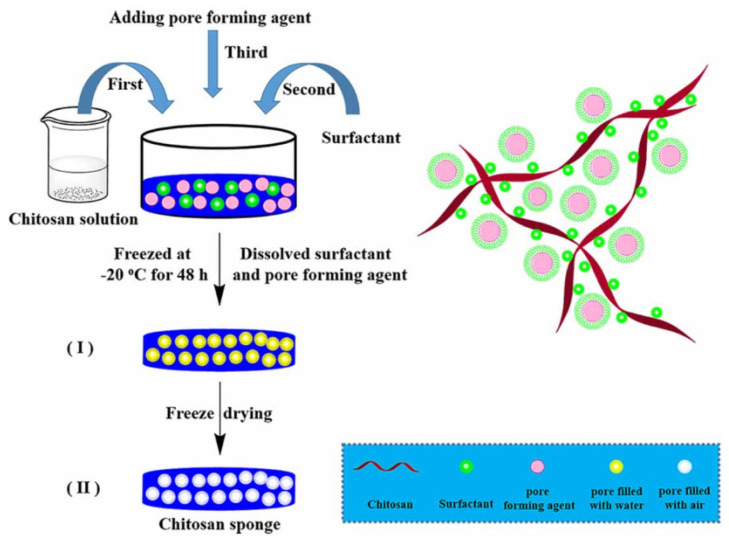
Schematic representation of the fabrication process of chitosan sponge and the interaction of its molecules. Reprinted with permission from Fan et al. [43]. Copyright 2020, IOP Publishing.

Sponges are porous solid systems, similar to scaffolds, but with a different manufacturing process. For the manufacture of chitosan sponges, the polymer is dissolved in an acidic or saline aqueous solution. A surfactant, usually sodium dodecyl sulfate, is then added while stirring at high speed to obtain a foam. A pore-forming agent may be incorporated into the foam at this point in the process. Finally, the system is lyophilized to obtain the chitosan sponge (Figure 4) [43]. The use of chitosan sponges in post-surgical procedures as hemostatic systems has been deeply studied [44]. These devices are valued for their biodegradability and antimicrobial activity and their ability to absorb large volumes of fluids. In addition, they can also be used as reservoirs for the release of antibiotics, such as doxycycline, thus improving their antibacterial activity [45]. Different modifications have been made to chitosan to improve the properties of these sponges. For example, hydrophobically modified chitosan sponges showed improved bleeding control compared to unmodified chitosan [46]; thiol-modified chitosan also showed excellent hemostatic performance [47], and alkylated chitosan sponges were able to rapidly absorb large volumes of water and blood [48]. It is also possible to develop mixed sponges, combining chitosan with other polymers, the combination of chitosan and gelatin being the most studied [49,50].

### 3.3. Meshes

Other devices widely used nowadays in surgery are meshes, replacing sutures in many cases, such as in hernia repair or pelvic floor construction. They are flexible networks formed by crosslinked fibers. Polypropylene meshes are the most common, being a resistant, economical, and non-resorbable material. However, there are still some cases of rejection due to foreign-body reactions and infections of the area [51]. These polypropylene-based meshes have been modified by including chitosan on their surface, which improves biocompatibility and antimicrobial properties, thus accelerating the healing process [52]. This synergy needs to be explored more deeply; another study evaluated the coating of polypropylene meshes with different concentrations of chitosan, but when they were implanted in rats, the adhesion and histopathological parameters were not modified [53]. It is also possible to obtain meshes of other materials. For example, a poly(N-isopropylacrylamide)/chitosan hydrogel mesh was able to form a swelling-resistant structure with improved adhesive properties [54].

### 3.4. Membranes

Similar to meshes, membranes or films can be manufactured. They are also flexible polymeric layers, but with a continuous structure instead of a fiber network. The standardized manufacturing technique for chitosan membranes involves the preparation of an acid solution of chitosan, which is freeze-dried after being poured into a mold. After this process, the membrane is treated with alkali to displace the acid used to dissolve the polymer and facilitate the polymerization of chitosan. Finally, the system is dried to obtain chitosan membranes [55]. Some authors have replaced the lyophilization step with the immersion of the membrane in liquid nitrogen for 10 s [56]. Chitosan acetate films were developed for biomedical applications. The influence of the inclusion of glycerol, oleic acid, and a mixture of them as plasticizers was evaluated. All of them became biodegradable films suitable for skin recovery [57]. Membranes can also be manufactured as multilayer films, allowing different materials to be combined and providing different properties to the membrane. For example, multilayer chitosan/poly(L-lactic acid) membranes combine the biocompatibility and cell-growth promotion of chitosan with the mechanical strength of poly(L-lactic acid) [58]. HemCon^®^ is a commercialized chitosan-based film that is recommended for preventing blood loss, thanks to its hemostatic properties, while providing an antibacterial barrier that avoids wound infection [59]. Similarly, CELOX^TM^ is a chitosan hemostatic dressing that has shown equivalence to HemCon^®^ in the control of bleeding [60]. Chitosan membranes can also be used in surgical procedures in combination with polypropylene mesh; they provide an antiadhesive barrier that prevents the adhesion of peritoneal tissue to the mesh [61]. There are also references to the use of chitosan membranes in guided tissue regeneration [62]. A collagen film impregnated with a layer of chitosan has been used as a barrier membrane for managing periodontal furcation, resulting in excellent biological acceptance and low gingival recession [63]. Chitosan membranes have also been developed for guided bone regeneration; these devices have antimicrobial properties and are capable of inducing angiogenesis, thus promoting bone regeneration in in vivo studies [64].

### 3.5. Hydrogels

Hydrogels have also been studied for surgical applications. These systems consist of a liquid phase, which generally comprises 90% of the formulation, trapped in a solid phase that gives the gel its structure [65]. This water content makes these systems highly biocompatible, and their soft consistency prevents damage to surrounding tissues. Chitosan hydrogels show similar mechanical properties to connective tissues, which favors tissue regeneration [66]. Three types of chitosan hydrogels for surgical applications are described in the literature: physically associated hydrogels based on the crosslinking of chitosan chains through hydrogen bonds or electrostatic interactions; coordination complex crosslinked hydrogels, which require metal ions to form covalent bonds with chitosan, making them less suitable for biomedical applications; and chemically crosslinked hydrogels that undergo irreversible gelation via covalent bonds but require modifications to the chitosan structure [67,68]. Chitosan hydrogels have been studied for different applications, such as hemorrhage control, dental and bone regeneration, and treatment of wound infections [69,70,71,72]. It is worth mentioning the possibility of developing thermosensitive hydrogels, which are capable of forming a gel after administration in response to increased temperature [7]. For example, a thermosensitive chitosan/gelatin hydrogel was developed for the sustained release of stem cells in therapeutic angiogenesis [73].

### 3.6. Nanofibers and Nanoparticles

Another formulation prepared from chitosan is nanofibers. The electrospinning technique allows the fabrication of chitosan nanofibers with a diameter of a few nanometers and, therefore, a large specific surface [66]. Porous meshes or scaffolds can be manufactured from the developed nanofibers [74,75].

Chitosan nanoparticles have also been developed for surgical applications. These formulations can be included in another system to enhance their activity. For example, melatonin-loaded lecithin–chitosan nanoparticles have been included in a hydrogel for topical administration as a wound-healing promoter. In vivo studies have shown the induction of angiogenic and fibroblast proliferation after the administration of nanoparticles [76]. Another example is the minocycline-loaded nanoparticles developed by Ma et al., which were included in a collagen/chitosan membrane for guided bone regeneration [64].

## 4. Surgical and Post-Surgical Applications of Chitosan-Based Devices

### 4.1. Nerve Regeneration

Nowadays, biomaterials are being widely studied for nerve regeneration. Among them, molecules of natural origin, such as collagen, silk, and chitosan, can be highlighted [77]. Chitosan has several properties that make it especially promising for this application. Its biocompatibility, its antibacterial activity, and its ability to interact with regeneration-associated cells and the neural environment, promoting axonal regeneration and less neuroma formation, are some of these properties [77,78,79].

Chitosan has gained special importance in the field of regeneration of peripheral nerves. Among other reasons, this is due to the ability to simulate the multilayer structure of these nerves in nerve conduits designed with artificial tissue, thanks to the physical and chemical properties of this polysaccharide [78]. However, it must be kept in mind that the degree of acetylation of chitosan can influence the mechanical properties and degradation time of the conduit and, consequently, the regeneration process [78,80]. Nerve-guiding conduits could help overcome problems of donor nerve availability and secondary injuries associated with autograft, which is the treatment of choice for peripheral nerve injuries [81]. Along these lines, in 2015, the FDA approved a chitosan-based nerve conduit under the tradename Reaxon^®^ Nerve Guide for bridging gaps of up to 26 mm in peripheral nerves [82]. It is a flexible nerve guide, the positive surface charge of which establishes an electrostatic interaction with negatively charged biomolecules and cellular components, contributing to nerve regeneration. Its hydrogel wall favrors the transport of oxygen and nutrients to the injured nerve, thus creating an optimal environment for Schwann cells. In addition, this nerve guidance inhibits the growth of fibroblastic tissue and scar formation [79].

Despite the above, researchers continue working to achieve the “ideal nerve tube”, which, according to Bąk et al. [80], would have the following properties: “biodegradability, porosity and permeability of the tube wall, presence of an inner scaffold made of fibers or filaments, the capacity to sustain cell livability and promote cell migration, the ability to secrete growth factors and electrical conductivity”. A large number of research works related to the use of chitosan in nerve regeneration can be found in the literature. Some of them are collected in Table 1.

The conduits currently available for nerve regeneration are hollow tubes, which are generally associated with poor recovery and difficulty in nerve extension due to scar formation. For this reason, research has focused on the inclusion of fillers (such as some of the examples in Table 1) and growth factors or the Schwann cells in them to enhance the regeneration process [92,93]. Chitosan is commonly used for the development of different vehicles to transport drugs or cells for nerve regeneration. For instance, verapamil-filled chitosan/polycaprolactone hybrid nanofiber conduits were tested in rats for regeneration of the transected sciatic nerve, suggesting a beneficial effect of calcium channel blockers on nerve recovery [92]. Regarding the enrichment of chitosan-based systems with cells or growth factors, Liu et al. demonstrated the efficacy of combining chitosan nerve conduits with nerve growth factor microspheres for the repair of injured facial nerves in rabbits [94], and Zhao et al. proposed a sponge-based hydroxypropyl chitosan/soy protein isolate composite conduit with bone marrow mesenchymal-stem-cell-derived Schwann cells for sciatic nerve regeneration in rats [95].

It is worth mentioning that chitosan has also been studied for the treatment of spinal cord injuries. Wang et al. formulated valproic-acid-labeled chitosan nanoparticles to combine the beneficial effects of valproic acid and chitosan nanoparticles for injured spinal cords [96]. Among the findings of this study were the promotion of functional recovery and tissue repair and the improvement of the integrity of the blood–spinal cord barrier. In contrast, Yang et al. developed a chitosan scaffold composed of graphene oxide that was transplanted into rats [97]. The results indicated that the scaffold promoted nerve cell growth, neuron migration, and tissue regeneration, in addition to showing better functional recovery than other chitosan scaffolds.

### 4.2. Bone Regeneration

Bone is an organ that can heal itself, thanks to a continuous remodeling process. However, this is only effective for small lesions (<8 mm); larger defects require bone substitutes and surgery [98]. Autografts, allografts, surgical reconstruction, and metal implants represent the traditional options for bone reconstruction, with autologous bone grafting being the treatment of choice [8]. Nevertheless, they are associated with issues such as the risk of disease transmission, rejection, or the need for repeated surgeries [99]. For this reason, tissue-engineered bone grafts are being studied as possible substitutes. According to Venkatesan et al., the implanted material must be biocompatible, osteoconductive, highly porous, and with good mechanical properties [100]. Furthermore, since bone is a highly vascularized tissue, implant vascularization is important for successful bone regeneration [98].

Given their biocompatibility and biodegradability, natural polymers are widely used materials for bone grafting. Among them, chitosan, collagen, silk fibroin, gelatin, cellulose, alginate, and starch can be highlighted [98]. Chitosan is an excellent candidate for bone reconstruction as it has antimicrobial properties, can generate porous structures suitable for cell growth and osteoconduction, and promotes osteoblast and mesenchymal cell proliferation and neovascularization in vivo. Furthermore, its structure is similar to glycosaminoglycans, a component of the bone extracellular matrix [98,100,101]. 

Chitosan has been formulated in different systems for bone tissue engineering, including scaffolds, sponges, hydrogels, micro-nanospheres, and membranes, among others [98,102]. According to Sukpaita et al., 3D biomaterial scaffolds are one of the three main pillars of bone tissue engineering, along with osteogenic stem cells and bioactive molecules such as growth factors or drugs. These scaffolds can be defined as implants capable of accelerating the formation of new bone integrated into the host bone without causing adverse reactions [102]. A large number of studies on chitosan-based scaffolds for this application can be found in the literature. It is usual to develop systems that combine chitosan with other compounds to improve properties such as osteoconductivity and mechanical properties and thus obtain materials that mimic natural bone as much as possible [100,103]. Among these reinforcing components, inorganic compounds such as hydroxyapatite stand out. Soriente et al. manufactured chitosan/hydroxyapatite composite scaffolds by the sol–gel method and subsequent lyophilization [104]. In these scaffolds, hydroxyapatite nanoparticles were embedded in the chitosan matrix. They observed that the higher the content of the inorganic component in the scaffold, the better the osteogenic differentiation of mesenchymal cells towards osteoblasts and the greater the anti-inflammatory response. 

The combination of chitosan with other polymers to obtain composite materials also frequently improves its applicability to bone regeneration [101]. Guo et al. manufactured electrospun nanofiber membranes made from collagen and chitosan [75]. The results of this work showed that these membranes had higher tensile strength, a more stable rate of degradation, and better in vivo results in repairing calvarial bone defects than electrospun membranes based only on collagen. 

Other strategies that have been proposed to improve the characteristics of chitosan-based systems for the repair of bone defects are the following:

- Inclusion of nanohydroxyapatite/chitosan microspheres in chitosan membranes, with better results than pure chitosan membranes in terms of mechanical properties [105].
- Incorporation of halloysite chitosan-modified nanotubes in thermosensitive hydrogels of chitosan/glycerophosphate, thus improving the mechanical properties and proliferation of the encapsulated stem cells with respect to the hydrogel [106].
- Functionalization of chitosan to give rise to derivatives such as sulfated methacrylate chitosan, which is associated with vascularization [107], and peptide-functionalized chitosan, which promotes a greater adhesion and proliferation of osteoblasts than chitosan [103].

Despite what has been described above, research works on systems based solely on chitosan for bone regeneration can also be found in the literature. For instance, Sukul et al. formulated sponges based on chitosan with different degrees of deacetylation and molecular weights and determined the influence of these parameters on the adhesion, growth, and differentiation of human osteoblasts [108]. 

Chitosan-based systems loaded with drugs, stem cells, or growth factors for bone regeneration have also been developed. Based on the osteogenic potential of simvastatin, Murali et al. formulated fatty-acid-modified electrospun chitosan membranes loaded with the drug [109]. Regarding the incorporation of stem cells, stem cells derived from human urine have been loaded onto hybrid scaffolds composed of biphasic sponges of calcium phosphate and chitosan [110]. Finally, porous chitosan scaffolds were loaded with protein growth factors for the repair of tibial defects in rabbits [111].

A specific application of chitosan within bone regeneration is periodontal bone repair. According to Xu et al., “periodontitis is a progressive infectious inflammatory disease, which leads to alveolar bone resorption and loss of periodontal attachment” [112]. Alveolar bone resorption causes serious and irreversible damage that cannot normally be repaired by physiological mechanisms. For this reason, bone grafts are often used to treat bone defects associated with periodontitis. One of the problems with conventional therapy is the proliferation of adjacent tissues into the defect before the migration of osteoblasts; this hinders bone regeneration and causes drawbacks such as implant encapsulation. Thus, guided bone regeneration (GBR) has emerged as a tool to prevent the ingrowth of these surrounding tissues, being one of the most effective methods in alveolar bone regeneration [113].

**Figure 5:**
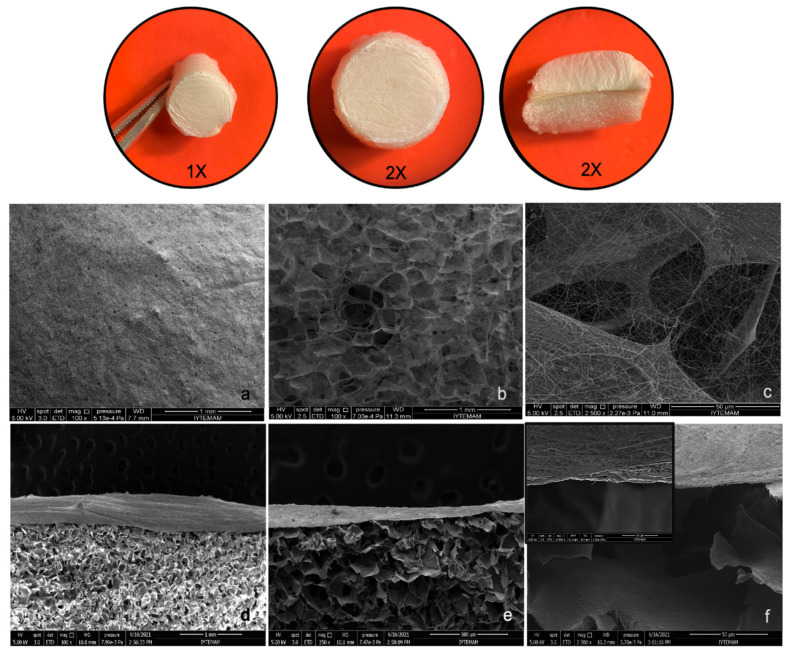
Stereomicroscopy images of bilayer membranes (1×, 2×) and SEM images of chitosan/PEO nanofiber coated porous layer surface (**a**–**c**) with 250×, 1000× and 2500× magnifications; cross-sectional view of bilayer structure (**d**–**f**) with 250×, 500× and 10,000× magnifications. Reprinted with permission from Tamburaci et al. [114]. Copyright 2021, Elsevier.

Among the systems studied for GBR therapy, membranes can be highlighted. Due to its properties, such as mucoadhesiveness, antimicrobial activity, and biocompatibility, chitosan has enormous potential for the development of GBR barrier membranes [62,113]. An example of the success of resorbable barrier membranes based on biopolymers is the work developed by Tamburaci et al. [114]. They fabricated a novel bilayer membrane composed of an upper layer of chitosan/polyethylene oxide, obtained by electrospinning, and a microporous sublayer of nanohydroxyapatite particles doped with chitosan/silica, obtained by lyophilization (Figure 5). The results showed that the first layer acted as a barrier, preventing the unwanted proliferation of fibroblasts, and the second layer participated in osteogenic activity at the site of the bone defect.

The incorporation of drugs in chitosan-based formulations represents a common strategy for periodontal bone repair. Specifically, collagen/chitosan membranes containing minocycline-loaded chitosan nanoparticles and minocycline-controlled release hydroxyapatite/chitosan composites have been studied to prevent infections associated with this type of bone regeneration [64,115]. In addition, based on the anti-inflammatory properties of metformin, composite scaffolds combining β-tricalcium phosphate, chitosan, and mesoporous silica SBA-15 have been loaded with this drug to repair alveolar bone defects in rats [112].

Periodontal regeneration includes not only the regeneration of alveolar bone but also the regeneration of cementum, periodontal ligament, and gingiva [62]. Thus, Varoni et al. proposed a three-layer scaffold based on chitosan for multi-tissue periodontal healing [116]. This system consisted of two porous compartments formed by chitosan of low and medium molecular weights, respectively, crosslinked with genipin and subsequently lyophilized, and a third compartment obtained by the electrochemical deposition of chitosan. The first and second compartments were intended for bone and gingival repair, while the third aimed to regenerate the periodontal ligament.

Finally, Yan et al. combined an enzymatically solidified chitosan hydrogel, the gelation of which was a pH-dependent process based on the enzymatic hydrolysis of urea with periodontal ligament cells for the repair of periodontal defects in rats [117]. Although the cells did not lead to the expected result, cell-free hydrogels showed great potential for this purpose in terms of functional ligament length.

It is worth mentioning that chitosan has also been used for two particular purposes within periodontal regeneration: augmentation of the maxillary sinus floor and treatment of periodontitis/peri-implantitis. Maxillary sinus floor augmentation is a surgery that consists of lifting the sinus membrane and placing a bone graft under it to increase bone support prior to a dental implant [118]. An example of the use of chitosan in the development of materials for this procedure is the work of Li et al., who prepared an injectable thermosensitive hydrogel composed of chitosan, β-sodium glycerophosphate disodium salt hydrate, and gelatin and loaded it with erythropoietin as a minimally invasive tool for maxillary sinus floor augmentation [119]. The system turned quickly into a gel at body temperature, allowing easy handling and a rapid formation of the final formulation once administered, and promoted new bone formation by intramembranous osteogenesis in vivo, thanks to the growth factor.

Periodontitis is defined as a pathology that causes inflammation of the connective tissue and loss of bone tissue surrounding the teeth; it is called peri-implantitis in the case of a dental implant [120]. They are usually associated with plaque biofilm dysbiosis [121] and bacterial biofilms on the implant surface [122], respectively. Treatment includes mechanical debridement and infection control with proper oral hygiene and patient care, up to surgery in the most severe cases [121,123]. Research in this field pursues new non-surgical treatments that are more effective than those currently available. The potential of chitosan for bone regeneration and its antimicrobial properties, among others, make it very interesting for the development of different systems for non-surgical periodontal treatment. Thus, a chitosan brush seated on an oscillating dental handpiece has been tested in a clinical trial for the debridement of residual pockets in the treatment of periodontitis compared to regular curettes. The results of this study indicated the potential of the chitosan brush for this application [121]. Zhou et al. fabricated hyaluronic acid/chitosan composite hydrogels loaded with dexamethasone for the treatment of peri-implantitis. These systems showed sustained drug release and adequate antibacterial and anti-inflammatory effects for the intended purpose [124].

### 4.3. Cartilage Regeneration and Viscosupplementation

Adult articular cartilage is an avascular tissue, which hinders its self-healing and leads to surgical procedures such as microfracture for its repair. However, these treatments are not always as successful as expected, so other tools are being studied for this purpose, such as biomaterials [125]. Its similarity with glycosaminoglycan and hyaluronic acid makes chitosan an interesting option for cartilage repair [126]. Moreover, chitosan is a polymer that shows great potential for viscosupplementation, which, according to Comblain et al., “is a process that aims to restore the normal rheological properties of synovial fluid” [127]. Hyaluronic acid, which is frequently used for viscosupplementation, has the disadvantage of remaining in the joint cavity for a short period. Based on what was described above, different works can be found in the literature on the use of chitosan for the repair of chondral and osteochondral defects (the latter involves a joint injury in which both the cartilage and the underlying bone are affected [128]) and viscosupplementation. Some of them are described below.

Abarrategi et al. studied the influence of molecular weight, deacetylation degree, and calcium content on the properties of chitosan for osteochondral tissue regeneration [126]. Chitosan-based porous scaffolds with different values of these parameters were manufactured and implanted in rabbits with osteochondral knee defects. Chitosan scaffolds with lower molecular weight, a lower degree of deacetylation, and intact mineral content turned out to be the most suitable among those evaluated for the regeneration of cartilage and bone in these defects. Akmeşe et al. compared a chitosan-based liquid scaffold with a hyaluronic-acid-based soft scaffold for the repair of osteochondral defects of the talus [129]. Although both cell-free scaffolds proved to be effective in treating these lesions in terms of cartilage repair, no differences could be established between them. Meanwhile, Hoemann et al. demonstrated the utility of chitosan–glycerol phosphate/blood clot implants for cartilage repair in microfracture defects in sheep [130]. These implants offered greater retention in the defect walls than normal clots at one hour postoperatively, which was attributed to the adhesive and thrombogenic properties of chitosan. Six months after the operation, the implants showed better results in terms of hyaline cartilage regeneration than microfracture alone. 

In addition, chitosan has also been used for the development of systems that load bioactive molecules, such as growth factors (for example, transforming growth factor-β1 [131,132]) and stem cells (such as synovial mesenchymal [133] or adipose-derived stem cells [134]) for cartilage regeneration. 

Regarding the use of chitosan for viscosupplementation, Scognamiglio et al. formulated hydrogels based on lactose-modified chitosan crosslinked with boric acid for the treatment of osteoarthritis [135]. One of the different compositions studied showed rheological properties similar to commercial products, so it was chosen for further characterization. This formulation was biocompatible and showed greater resistance to degradation than the hyaluronic-acid-based samples, which translated into greater stability after administration. The selected hydrogel was also associated with the inactivation of ROS, which are involved in osteoarthritis.

### 4.4. Soft Tissue Regeneration

Soft tissue engineering can be described as the use of biomaterials for the restoration of the biological function of defective or damaged soft tissues, such as skin, mucosa, or cornea, among others [136]. Wound healing occurs naturally through a number of biological processes, including inflammation and cell proliferation. Soft tissue engineering can improve the efficacy of these processes. In the case of severely damaged tissue that cannot be repaired by organic responses, biomaterials can also replace that tissue [137]. These biomaterials must be biocompatible, have antibacterial activity to protect the damaged tissue from infection, absorb liquids, and allow gas permeation. Moreover, they must have optimal mechanical strength and should provide a favorable microenvironment for new cells [136,137]. 

Chitosan has several advantages for soft tissue regeneration. First of all, it meets all the requirements mentioned above for biomaterials intended for soft tissue engineering. It also has other characteristics, including its slow rate of degradation by different enzymes into oligomers and, ultimately, into N-acetylglucosamine, a common amino sugar that induces and enhances the natural healing process due to its anti-inflammatory properties [138]. Moreover, chitosan has antifungal, antimicrobial, immunostimulant, and hemostatic properties, features that are related to the optimal restoration of damaged tissue [66].

Chitosan can be formulated in hydrogels and in 2D and 3D materials, and each of them has different possibilities. To begin with, hydrogels are soft, flexible systems that can be applied to mimic soft tissue without damaging adjacent structures. Two-dimensional materials include thin films or porous membranes, which can be easily used to cover small to large damaged surfaces (such as skin wound healing), protecting them from external agents and enhancing natural healing. Lastly, 3D scaffolds can be manufactured with tunable porosities and enhance regeneration by inducing matrix restoration, allowing the diffusion of nutrients and gases in the regeneration of soft systems [66]. Thanks to the aforementioned properties of chitosan, it has been widely explored for soft tissue regeneration (Table 2).

#### 4.4.1. Skin and Mucosa Wound Healing

Madrazo-Jiménez et al. conducted a clinical trial to evaluate the effectiveness of a chitosan hydrogel containing allantoin, dexpanthenol, and chlorhexidine as a wound healing system [146]. The use of this gel significantly improved the healing process in the buccal mucosa after the extraction of third molars. As an example of chitosan-based 2D materials, Zaitun Hasibuan et al. mixed cellulose nanofibers, chitosan, and silver nanoparticles in a wound-healing dressing [147]. Based on the results of the study, the authors were able to conclude that this system is a non-hemolytic material with strong antibacterial properties, making it an interesting candidate for future in vivo studies. Ruprai et al. also developed 2D structures as adhesives. These systems, based on chitosan and loaded with L-DOPA, were obtained by lyophilization and showed a photochemical binding capacity to tissue when treated with green light. Interestingly, this approach overcomes one of the main limitations of glues and patches, as the porous structure of freeze-dried chitosan allows the efficient permeation of gases and substances, thus enhancing the wound healing process [148]. Another approach was proposed by Zhu et al., who developed flurbiprofen-grafted chitosan/alginate composite-based 3D scaffolds for subcutaneous implantation [135]. These scaffolds were prepared by the lyophilization of a previously obtained gel and then grafted with crosslinkers and flurbiprofen. The obtained scaffold exhibited a homogeneous porous structure with optimal mechanical properties. In addition, the presence of flurbiprofen was synergized with the structure and characteristics of the scaffold, enhancing cell adhesion and proliferation.

#### 4.4.2. Cornea Damage

Corneal damage is the cause of vision loss in 10 million people yearly; 12 million people have already gone blind. Corneal damage leads to the opacity of the cornea due to the formation of scar tissue. Nowadays, synthetic or allogenic implants are the most widely used options for their treatment, but adverse effects such as high rejection rates frequently occur, and the shortage of donors generates long waiting lists. In the search for alternatives, chitosan has emerged as a good option [149,150].

Although chitosan has a number of advantageous properties, the films obtained by the solvent casting method have poor mechanical properties. According to this, Tayebi et al. prepared chitosan/poly-ε-caprolactone composite films loaded with chitosan nanoparticles as a vehicle for corneal endothelial cells [151]. The authors found that chitosan nanoparticles improved biocompatibility and surface properties without affecting the transparency of the systems, which is a key requirement for this application. Corneal epithelial cells were also able to adhere to the membrane, survive, and proliferate adequately. This membrane could, therefore, be an option for the future in the treatment of corneal damage.

**Figure 6:**
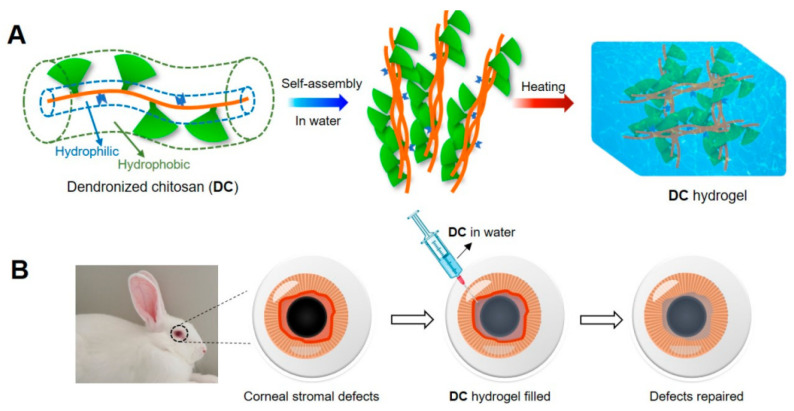
Schematic illustration for the formation of dendronized chitosan (DC) hydrogels and the application for corneal stromal defects. (**A**) Cartoon presentation of DCs featured with radial amphiphilicity and their self-assembly in water to form fibrous bundles and the instant formation of hydrogels via heating around physiological temperature. (**B**) Injection of DC solution into the corneal stromal defects of a rabbit model, in situ formation of a hydrogel filler, and defects repairing. Reprinted with permission from Feng et al. [152]. Copyright 2021, American Chemical Society.

Another innovative approach was described by Feng et al., who developed transparent thermogelling scaffolds using oligoethylene glycol-based dendronized chitosan [152]. Interestingly, the gel point of these systems can be easily adjusted. These transparent hydrogels were shown to be capable of promoting the migration and proliferation of keratocytes, with a positive effect on corneal regeneration in a rabbit animal model (Figure 6).

#### 4.4.3. Gastric Ulcer

Gastric ulcer can be described as a pathological condition that occurs when the gastric epithelium is damaged by excessive inflammatory erosion, usually leading to epigastric pain and gastric mucosa bleeding. Different factors can increase the risk of gastric ulcers, such as the excessive consumption of alcohol or non-steroidal anti-inflammatory drugs, as well as bacterial infections such as *Helicobacter pylori* [153,154].

In order to improve the actual treatments for gastric ulcers, Maeng et al. designed an endoscopic chitosan hydrogel loaded with epithelial growth factor, given that it binds to a receptor that initiates a series of responses that accelerate tissue regeneration [155]. The authors evaluated this gel using in vitro (wounded cell monolayer) and in vivo (GI ulcers in rabbits and micro-pigs) models of ulcers. Histological analysis allowed them to conclude that this formulation significantly reduces the time required for the complete healing of gastric ulcers.

#### 4.4.4. Chronic Tympanic Membrane Perforation

Most tympanic perforations heal spontaneously in 7 to 10 days, thanks to epithelial migration, fibroblastic activity, and vascular proliferation. However, cases in which healing does not occur spontaneously within 3 months are called chronic tympanic perforations, which require intervention for recovery [156]. Although there are surgeries with high efficacy (>90%), there are some drawbacks, such as the high cost or the risk of anesthesia. For this reason, new biomaterials for non-invasive applications have recently been studied, among which chitosan stands out: it is highly biocompatible and allows cell adhesion and wound healing. In addition, it has good mechanical properties and is antibacterial [157]. For this reason, chitosan has been shown to be effective in improving the healing of tympanic perforations [158]. In this context, Kim et al. prepared 3D chitosan porous scaffolds and evaluated their structural and mechanical properties, as well as biocompatibility and healing effects, in an in vivo model [159]. These scaffolds allowed the regeneration of the perforated tympanic membrane through cell migration, epidermal connective tissue, and mucosal restoration. However, to improve the properties of chitosan scaffolds, Seonwoo et al. prepared and evaluated chitosan scaffolds loaded with epithelial growth factor [157]. This factor may enhance healing by aiding the migration of fibroblasts, endothelial cells, and vascular cells. Their results allowed the authors to demonstrate that the loaded scaffold improved the ability of raw chitosan in terms of cell viability and in vitro wound-healing rate. This approach is not as effective as surgical methods, but it is an alternative option for patients with less severe tympanic perforations, or it could even be combined with surgery to improve results.

### 4.5. Sutures

Sutures, medical devices intended to stabilize wounds to accelerate their healing, have traditionally been made from non-bioabsorbable substances, although bioabsorbable materials have gained attention in recent years. These devices can be made up of a monofilament or a set of twisted or braided filaments. There are some requirements for sutures, including good mechanical properties and high biocompatibility [34,160]. Among the most frequent problems attributable to the sutures used today, the frequent infection of the tissues adjacent to the sutured wounds has recently attracted the attention of researchers. There are several strategies proposed to reduce the incidence of infections, such as the incorporation of an antibacterial drug (such as triclosan) or the use of antibacterial materials [161]. It is at this point that the use of chitosan is of interest since, in addition to its known antibacterial, hemostatic, and bioabsorption properties, it has also been shown to be capable of improving and accelerating the healing process [160]. Two possible strategies have been proposed: the design of chitosan fibers, alone or in combination with other polymers and drugs, and the coating of fibers with chitosan.

As an example of the former, Tan et al. prepared electrospun monofilaments with chitosan of different molecular weights and deacetylation degrees [34]. These devices were then characterized through various techniques, including surface morphology, mechanical properties, swelling ratio and in vitro degradation, and cytotoxicity, among others. The authors found out that chitosan with a molecular weight of 1.2 × 10^6^ g/mol and a degree of deacetylation of 85% allowed them to manufacture fibers with outstanding properties in terms of mechanical strength and biocompatibility.

Another approach was proposed by Costa da Silva et al., who loaded N-acetyl-D-glucosamine into chitosan filaments through the wet-spinning method [160]. N-acetyl-D-glucosamine has proven effective in accelerating the recovery of the epithelium. Several characterization techniques were applied, including SEM, evaluation of mechanical properties and in vitro biodegradations, drug release, and cytotoxicity. These fibers rapidly degraded due to the presence of N-acetyl-D-glucosamine, although the mechanical properties were somewhat worse compared to pure chitosan-based filaments. However, its mechanical performance was still better than that required by the U.S. pharmacopeia. Furthermore, drug release was maintained and biocompatibility was verified.

Regarding chitosan-coated fibers, Mohammadi et al. prepared chitosan/hyaluronic acid-coated nylon fibers using the layer-by-layer technique [162]. The two polymers selected for the coating were chosen due to their ability to form polyelectrolyte complexes. The authors found that coating with the polyelectrolyte complex leads to an improved growth rate of Vero cells on the coated filament. This, coupled with the antibacterial activity of the coating, makes this approach a good option for the development of next-generation sutures.

### 4.6. Hemostasis

Hemostasis is the biological process of maintaining blood fluidity in intact blood vessels and causing blood to coagulate when a vessel is damaged to prevent bleeding [163]. This process may be impaired in some diseases, such as hereditary hemophilia, where a coagulation factor deficiency prevents the body from stopping bleeding [164]. In surgical procedures, it is common to make incisions that cause tissue or blood vessels to rupture. Control of bleeding is vital in these situations to ensure patient survival. For this, the procoagulant effect of chitosan has been studied. Although the mechanisms of chitosan to stop bleeding are still uncertain, it was evaluated how the physicochemical properties of chitosan and the changes in its structure modify this characteristic. Thus, it was concluded that chitosan with a lower degree of deacetylation and higher molecular weight improves blood coagulation. Moreover, chitosan derivatives obtained by treating the polymer with acids show an enhanced procoagulant effect [165].

Considering this, different biomaterials based on chitosan have been developed to be used in the control of hemorrhage in surgical procedures. An example of this is chitosan hemostatic sponges, which combine the anticoagulant activity of chitosan with the high porosity and fluid absorption capacity of this device. Absorbable chitosan-based sponges of different degrees of deacetylation were fabricated and tested in vivo in a liver hemorrhage model. From the results, sponges based on chitosan with a degree of deacetylation of 40% were the ones that showed the best hemostatic effect. The blood and tissue compatibility of the device was also notable. The biodegradability of the system was remarkable, with visible degradation in the first week after implantation and the complete absence of the sponge after 4 weeks [44]. Sponges can also be prepared from a combination of chitosan and other polymers. The combination with gelatin is frequently used as these polymers can combine their advantages. Thus, a chitosan/gelatin sponge was prepared to be used as coagulation-promoting material and compared with sponges based on only one of these polymers. Composites were evaluated in rabbits for the coagulation of ear artery and liver injuries. The coagulation process was accelerated with the use of these sponges since platelet aggregation was promoted, which reveals the potential of these sponges to be used in surgical hemorrhages [49]. Some modifications of chitosan have been synthesized, particularly for use as procoagulant materials. Cheng et al. prepared a marine collagen peptide grafted with carboxymethyl chitosan and then manufactured sponges from this material. Both in vitro and in vivo tests revealed the procoagulant effect of these devices, which could be useful for hemorrhage control [166]. Du et al. manufactured sponges based on microchanneled alkylated chitosan. The main advantage of this modification is the increased blood absorption capacity due to the increased porosity of the developed system. When evaluated in vivo in rat and pig liver perforations, the use of this sponge was shown to be superior in hemostatic capacity to other commercial gauzes and to gelatin-based sponges [48]. Another example of sponges based on chitosan derivatives is the thiol-modified chitosan sponges developed by Wu et al. These authors included silver nanoparticles in the prepared system to increase the antibacterial activity of the device. As a result, the sponge has both hemostatic and antimicrobial performance, accelerating wound healing and avoiding infections [47].

**Figure 7:**
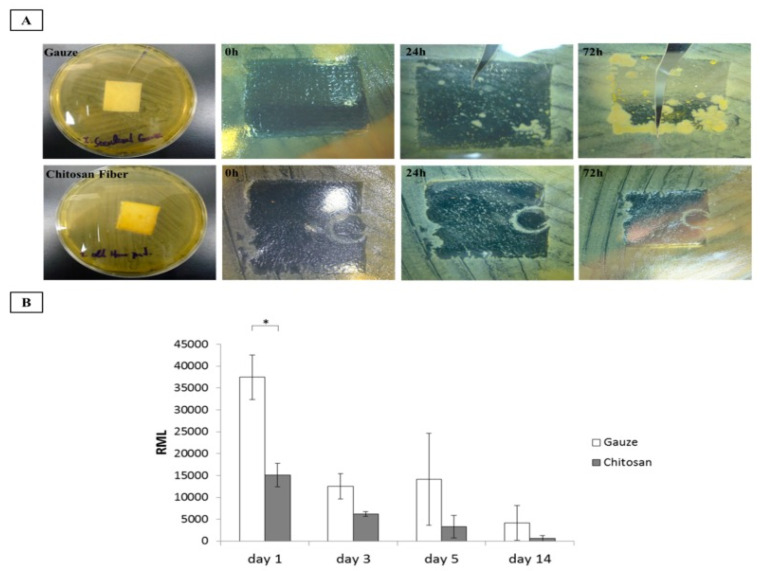
Antimicrobial activity evaluation. (**A**) In vitro. (**B**) Adenosine triphosphate (ATP) assay of microbial proliferation in patients with surgical wounds, * *p* < 0.05. Reprinted from [167] under the terms of the Creative Commons Attribution License 4.0 (copyright 2019 Wang et al.; doi:10.3390/polym11111906).

The efficacy of chitosan sponges has also been compared to chitosan fiber dressings. Both systems are highly biocompatible, but while the sponges offer a higher fluid absorption capacity, chitosan fiber dressings show the most rapid hemostatic effect. Moreover, this device also serves as an inhibitor of microbial proliferation (Figure 7) [167]. The advantages of chitosan dressings compared to traditional cotton surgical gauzes have been verified by different authors, the antimicrobial and procoagulant characteristics of chitosan being the ones that give this material promising properties for its use as a first-line dressing in the management of surgical hemorrhages [168]. Another study evaluated the efficacy of chitosan-based dressings in controlling hemostasis after dental extractions in patients receiving oral antithrombotic therapy. Again, the efficacy of this material was compared to the use of cotton gauze dressings. Not only was the mean time for hemostasis significantly reduced with chitosan-based dressings, but the incidence of adverse events—dry socket and pus discharge—was also notably lower [169]. The combination of fibrin glue and chitosan dressings has also been explored to improve the hemostatic process in patients with hemophilia. The conclusion of the study was that, although the use of these systems is not always necessary to achieve hemostasis, they have a particularly useful synergistic effect in surgical procedures with excessive bleeding [170].

It is not only sponges and dressings that have been developed as chitosan-based systems for hemostasis control. Logun et al. developed a hydrophobically modified chitosan foam, which was tested in rats with non-lethal liver excisions. A chitosan-based foam, a hydrophobically modified chitosan-based foam, and a control using fibrin sealant were evaluated. The expandable foam was injected into the damaged area, and the animals were observed for 6 weeks, after which they were sacrificed and the injury sites visualized microscopically. The developed systems proved to be safe and well-tolerated by animals. Both chitosan and chitosan-modified foams exhibited faster biodegradability after application than fibrin sealant and minimal tissue adhesion [171]. Chitosan-based tampons have also been developed as a strategy to prevent vaginal bleeding after loop electrosurgical excision procedures. The presence of hemorrhages in the 2 weeks following the surgical procedure was evaluated and compared to the use of a general tampon. Bleeding, as well as vaginal discharge and abdominal pain, was significantly reduced in the group of women who used the chitosan tampon, proving the effectiveness of this material in wound healing [172].

Another formulation that is being evaluated for hemostasis is chitosan gels, with several examples of this application in the literature. For example, the use of a chitosan gel as a coagulation promoter after endoscopic sinus surgery has been extensively studied, both in animal and human models [173,174,175]. This gel not only improves hemostasis but also prevents adhesion between tissues. A modified biodegradable chitosan hydrogel developed by Fang et al. was able to achieve a rapid sol–gel transition after injection and improved adhesion to biological tissues. In addition, the self-contracting property of this hydrogel provides an advantage for wound healing over other chitosan-based hydrogels [176]. Finally, it is worth mentioning that it is not only chitosan that has been studied to prepare these hemostatic hydrogels but also its combination with other compounds. As an example, a hydroxypropyl chitosan/soy protein isolate hydrogel was developed by Zhao et al. for hemorrhage control. This formulation was able to effectively reduce bleeding in an animal model [69]. Additionally, chitosan has the ability to open cell tight junctions, which may be useful in improving drug delivery. This feature can also be applied to hemostatic control by including hemostatic drugs in the chitosan-based formulation. For example, the inclusion of tranexamic acid in a mixed chitosan–dextran gel applied after endoscopic sinus surgery was used to control bleeding [177].

However, it is not only surgeries that require adequate hemostatic control. When a patient suffers successive punctures in a certain area, as can occur in chronic hemodialysis patients, they may suffer from acquired coagulopathy and excessive clotting time after needle removal. Chitosan-based systems have also been explored for these situations. Thus, Misgav et al. evaluated the potential of chitosan-based pads to shorten the time needed to control bleeding in these patients. The application of the pad on the puncture site proved to be effective in significantly reducing the time required to seal the hemorrhage—it was reduced from 18.5 to 3 min in arterial access and from 13.2 to 2.8 min in a venipuncture site [178]. The InnoSeal hemostatic pad is a commercially available catecholamine–chitosan pad, capable of significantly reducing hemostasis time after cardiac catheterization compared to other available pads [179]. Similarly, the Clo-Sur^Plus^ Radial^TM^ pad is another chitosan-based hemostatic pad evaluated for sealing bleeding after transradial arterial access. Although its efficacy was first compared with mechanical compression, it was concluded that the combination of both strategies is the most appropriate resource for bleeding control, with the chitosan pad being responsible for minimizing complications at the access site [180].

### 4.7. Other Surgical Applications of Chitosan

Although the use of chitosan for tissue regeneration and homeostasis control has been deeply explored, the innate properties of this polymer have made it a candidate for several alternative applications. As mentioned before, it has a slightly antimicrobial activity that becomes highly useful in surgically implemented devices. Titanium coating with chitosan by covalent bond was performed through a coupling agent (triethoxysilylpropyl succinic anhydride). This chitosan-coated titanium alloy proved to be active in reducing the growth of *Staphylococcus aureus* and *Escherichia coli* on the surface of the device, which could be useful in preventing infections after implantations of prostheses or devices made of this material [181]. In addition, chitosan can enhance the antimicrobial activity of drugs. With this in mind, nanofibers with a Nylon-6 core and a chitosan/polyethylene oxide shell structure, including two antimicrobial compounds, were fabricated; 5-chloro-8-quinolol was incorporated in the shell and poly(hexanide) in the core. Surgical meshes based on nanofibers were developed from this material with the purpose of use as meshes for hernias. This material showed greater in vitro activity against *Staphylococcus aureus* and *Pseudomonas aeruginosa* compared to the use of free drugs [182]. Similarly, implantable chitosan sponges loaded with cefuroxime, ciprofloxacin, and vancomycin were evaluated in terms of their antibacterial potential. It was observed that the high aqueous solubility of vancomycin made the system highly hydrophilic; thus, the sponge underwent rapid degradation, rendering it useless as an implantable device. However, the sponges loaded with cefuroxime and ciprofloxacin achieved a sustained release of the drug, with high tissue concentrations of the active ingredient and lower plasma levels of antibiotics [183]. This did not rule out the inclusion of vancomycin in chitosan-based biomaterials. Foster et al. managed to develop a vancomycin-loaded chitosan film, intended to prevent infections at surgical sites. The combination with chitosan was shown to improve the antimicrobial efficacy of the antibiotic. Furthermore, the adhesive properties of chitosan were enhanced through laser irradiation of the film after implementation. This adhesiveness would be highly useful in facilitating wound healing [12]. 

In fact, the use of chitosan films as a surgical adhesive was deeply studied by this research group. The authors demonstrated the efficacy of this system as an adhesive for ovine intestine regeneration [184]. Subsequently, the laser-activated chitosan thin film developed by these researchers (SurgiLux), with positive results tested both in vitro and in vivo on several tissues, was approved by the FDA for use as a post-surgical adhesive [185]. Different strategies to improve the adherence of chitosan biomaterials to biological tissues have also been evaluated. For example, the use of microbial transglutaminase was able to accelerate the binding of chitosan to different biological tissues (cardiac, dermal, and hepatic) and to polydimethylsiloxane-based devices [186]. Similarly, the mixture of chitosan with oxidized dextran was used to prepare a biocompatible and biodegradable injectable adhesive, with an adhesiveness of 4–5 times higher than that of fibrin glue [187]. Finally, it is noteworthy that despite the proven adhesive properties of chitosan, the chemical modification of this polymer has also been studied to reduce its adhesiveness. This is also useful in post-surgical applications as these chitosan-based antiadhesive materials could prevent peritoneal adhesion after abdominal surgery [188].

Aussel et al., who developed chitosan hydrogels for small-diameter vascular grafts, studied another application of chitosan materials. The mechanical properties of the fabricated device proved to be strong enough to withstand the intended application [189]. In addition, the implantation of two chitosan tubes as carotid grafts in sheep demonstrated that this polymer could be sutured without breaking, maintaining arterial pressure without flow obstruction [190]. Kang et al. have evaluated the use of hydroxyapatite–chitosan patches for the obliteration of the mastoid cavity. It was assessed in the tympanic cavity of rats and was shown to be superior to homologous cartilage and bone cement in mastoid obliteration [191]. Another curious application of chitosan is the use of chitosan/carbon nanotubes in hemoperfusion, as studied by Zong et al. Nanocomposite beads based on these nanotubes were fabricated, and their bilirubin adsorption capacity was evaluated. These chitosan-based materials showed their potential to be used in blood purification [192]. Finally, it is worth mentioning that the use of chitosan has also reached diagnostic applications. Ghosh et al. developed a derivative of chitosan through the crosslinking of iodinated 2,5-dimethoxy-2,5-dihydrofuran, thus becoming a radiopaque polymer used to manufacture microspheres with in vivo contrast properties, suitable for use in clinical diagnostics of the gastrointestinal tract [193].

## 5. Concluding Remarks

Chitosan is one of the most studied biopolymers for surgical applications due to its biodegradability, biocompatibility, and absence of toxicity. Its characteristic pH-dependent solubility provides the molecule with positive charges due to the protonation of amine in acidic media, which favors the formation of polyelectrolyte complexes and allows its union with mucosal tissues. The versatility of this polymer is reflected in the great variety of formulations and devices that have been manufactured, highlighting, in particular, scaffolds, sponges, membranes, and hydrogels. The developed materials take advantage of the natural bacteriostatic, fungistatic, hemostatic, and analgesic properties of chitosan.

The use of chitosan-based systems—mainly scaffolds—has been deeply explored for tissue regeneration. The clearest example is bone regeneration, where chitosan scaffolds provide biodegradable support for cell growth. Similarly, nerve tissue, cartilage, and various soft tissues can also be regenerated using chitosan-based systems. This application of chitosan would be really promising as it could allow, or speed up, the regeneration of tissues that cannot heal themselves. Another interesting application of chitosan in the manufacture of surgical material is chitosan-based dressings for hemostasis control. Several studies have shown chitosan’s improved ability to control bleeding and prevent infection compared to traditional cotton surgical gauze. Therefore, it is expected that, in the future, chitosan may be the material of choice for the development of these gauzes. The antimicrobial properties of chitosan have also been used to manufacture surgical meshes or sutures that could effectively prevent post-surgical infections, which, nowadays, are a frequent complication that increases treatment costs and mortality. Based on the multiple applications that derive from the innate properties of this polymer, it is expected that chitosan will soon be the material of choice for surgical applications.

## Figures and Tables

**Table 1:** Studies of chitosan-based systems for nerve regeneration.

Chitosan Form/System	Treated Nerve	Main Results	Reference
Chitosan–selenium biodegradable nanocomposite conduit	Sciatic	Number and diameter of myelinated fibers significantly higher against chitosan	[83]
Chitosan tubes with different degrees of acetylation	Sciatic	Intermediate degree of acetylation as the best choice in terms of degradation and regeneration efficacy	[84]
Laser-activated chitosan	Posterior tibial	Good functional recovery and tensile strength with laser-activated chitosan	[85]
Double-layer composite hydrogel conduit based on chitosan	Sciatic	Significant regeneration against chitosan hollow conduit and repair ability comparable to autologous transplantation when loaded with 7,8-dihydroxyflavone	[86]
Chitosan gel absorbed into Spongostan^®^	Facial	Positive effect of chitosan gel in nerve healing and better results when combined with platelet-rich plasma	[87]
Corrugated chitosan-film-enhanced chitosan nerve guides	Median	Accelerated functional recovery and thicker myelin sheats against other nerve guides	[88]
Aligned chitosan nanofiber hydrogel grafted with peptides as conduit filler	Sciatic	Enhanced nerve regeneration, secretion of neurotrophic factors, vascular penetration, and functional recovery than other conduits	[89]
Chitosan functionalized magnetic nanoparticles	Sciatic	Nerve outreach without surgical intervention and better functional outcome versus without treatment	[90]
Polycaprolactone/chitosan–hydroxyapatite hybrid implants	Peripheral	Possibility of controlling the diffusion of oxygen and nutrients and invariable mechanical properties for up to 28 days	[91]

**Table 2:** Some recent advances in chitosan-based materials for soft tissue regeneration.

Chitosan Form/System	Purpose	Main Results	Reference
Chatechol-conjugated chitosan patch	Oral mucositis	Mucoadhesive patches with enhanced healing properties through sustained release of triamcinolone acetonide.	[139]
Carboxymethyl chitosan/alginate-plantamajoside hydrogel	Burn wound skin	Reduces inflammation, increases collagen deposition, promotes cell migration and proliferation, and accelerates skin scald repair.	[140]
Cotton fabrics coated with carboxymethyl chitosan	Damaged skin	Antibacterial properties against *S. aureus* and *E. coli* and accelerated reepithelization.	[141]
Poloxamer 407/methylcellulose chitosan thermosensitive gel	Cornea damage	Good spreading ability, mucoadhesion, and ocular biocompatibility; accelerated corneal healing.	[142]
Chitosan patches	Tympanic perforation	More effective than spontaneous healing in tympanic regeneration.	[143,144]
Chitosan-coated filaments	Surgical suture	Significant reduction of biofilm formation.	[145]

## Data Availability

Not applicable.
